# Correction: Understanding the role of Shroom3 in the developing mouse myocardium

**DOI:** 10.1371/journal.pone.0341029

**Published:** 2026-01-13

**Authors:** The PLOS One Staff

## Notice of Republication

This article was republished on December 22, 2025, to correct an error in the author list: Thomas A. Drysdale was erroneously displayed as Thomas A. Drysda e. The publisher apologizes for the errors. Please download this article again to view the correct version. The originally published, uncorrected article and the republished, corrected articles are provided here for reference.

## Supporting information

S1 File Originally published, uncorrected article. (PDF)

S2 File Republished, corrected article. (PDF)
