# Supplementary figures and images for: Understanding the role of Shroom3 in the developing mouse myocardium
Source: PLoS One. 2025 Sep 8;20(9):e0331583. doi: 10.1371/journal.pone.0331583 (PMC12416694; doi:10.1371/journal.pone.0331583)

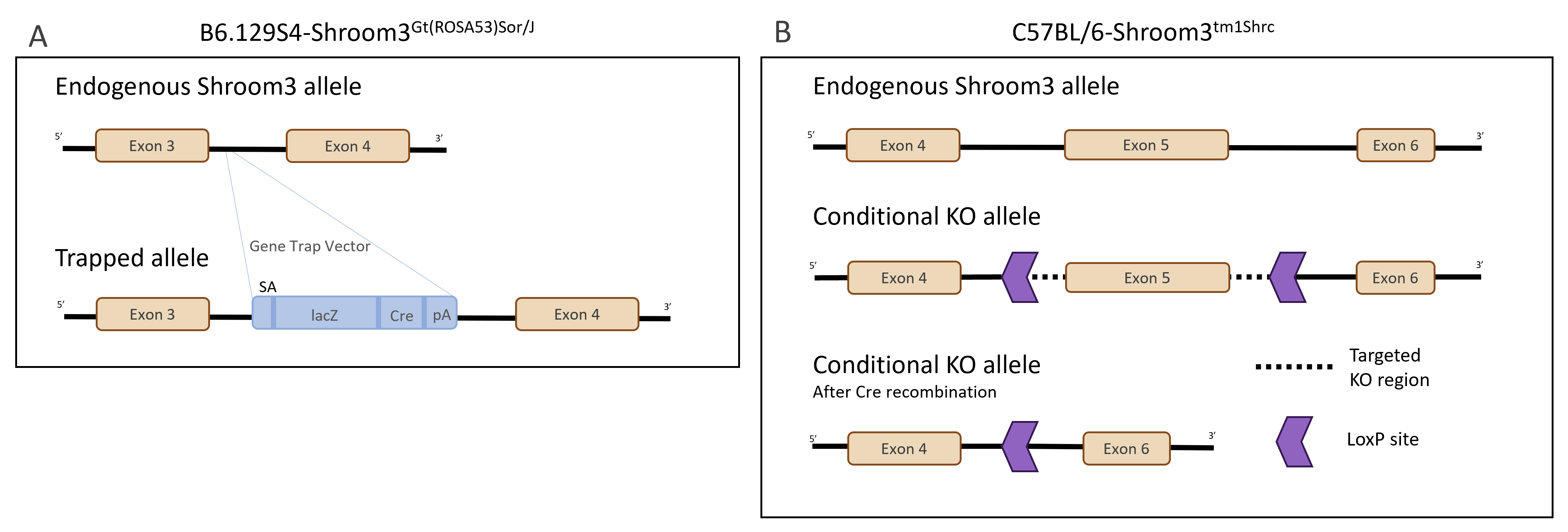

Supplement: S1 Fig — A) Schematic of Shroom3 gene trap line B6.129S4-Shroom3Gt(ROSA53)Sor/J. The inserted cassette includes a Splice Acceptor site (SA), an E.coli lacZ, Cre recombinase (Cre), and a polyadenylation sequence (pA). This inserted cassette is under control of the endogenous Shroom3 promoter. Insertion between exon 3 and exon 4 prevents proper mRNA formation, preventing functional protein from being made. B) Schematic of the novel floxed Shroom3 allele, created by Cyagen. LoxP sites were inserted to flank exon 5 of the endogenous Shroom3 gene. Upon recombination, the constitutive knockout allele was designed to produce a null allele. (TIF) [file pone.0331583.s001.tif]

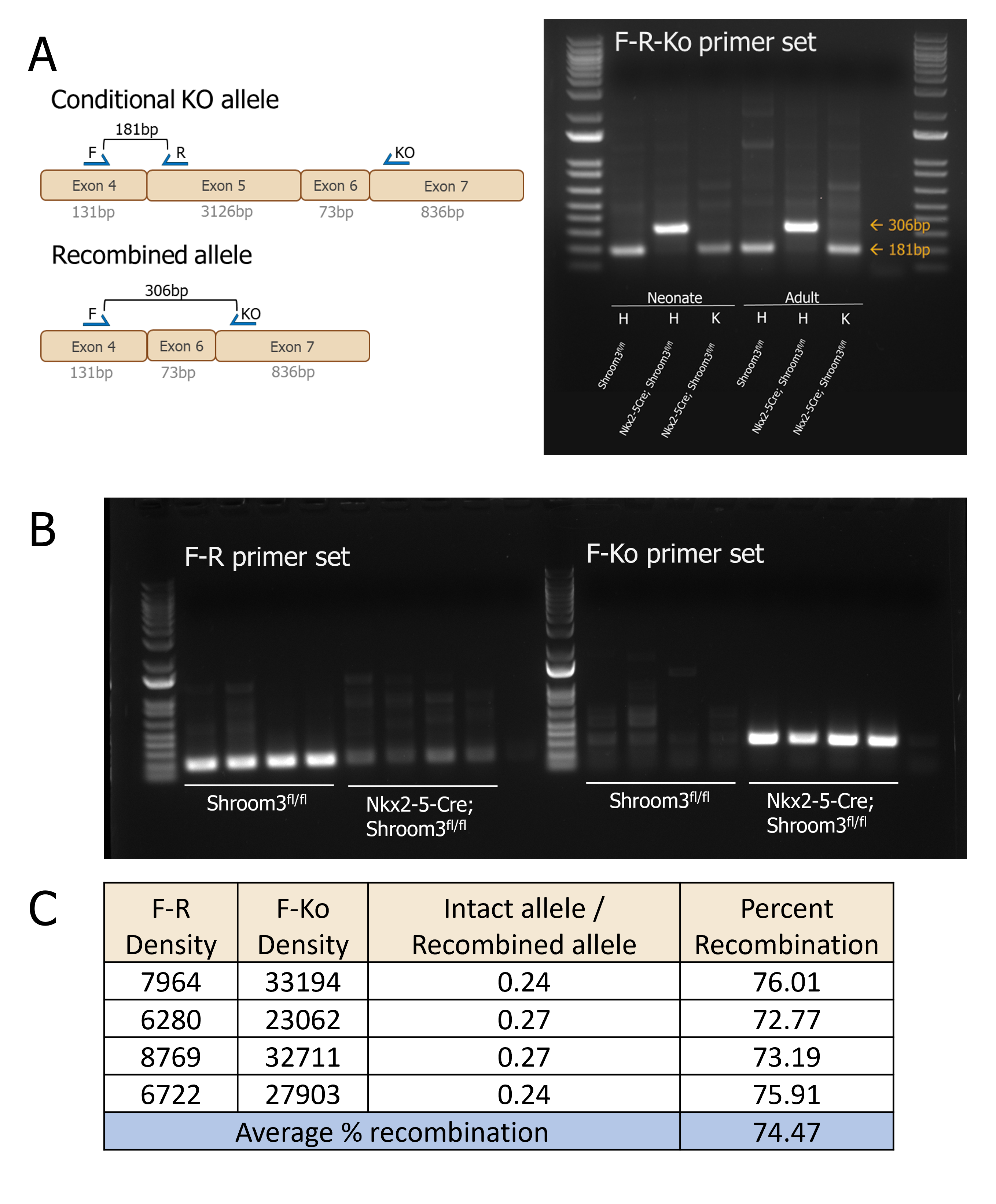

Supplement: S2 Fig — A) Primers were designed to detect recombination of the floxed Shroom3 allele recombination. Forward and reverse primers create 181 bp band (lower band, right gel). Forward and knockout primers create 306 bp band (upper band, right gel). Samples from neonate and 10m adult mouse heart, with kidney for tissue specificity B) cDNA samples from E18.5 hearts. Genotypes for samples are indicated. Samples were run with F-R primer sets and F-Ko primer sets. C) Average band density from F-R primer set, compared to band density from F-Ko. n = 4. F = Forward, R = Reverse, Ko = Knockout, H = Heart, K = Kidney. Raw gel images and lane annotations can be found at Open Science Framework (https://doi.org/10.17605/OSF.IO/NUMF2). (TIF) [file pone.0331583.s002.tif]

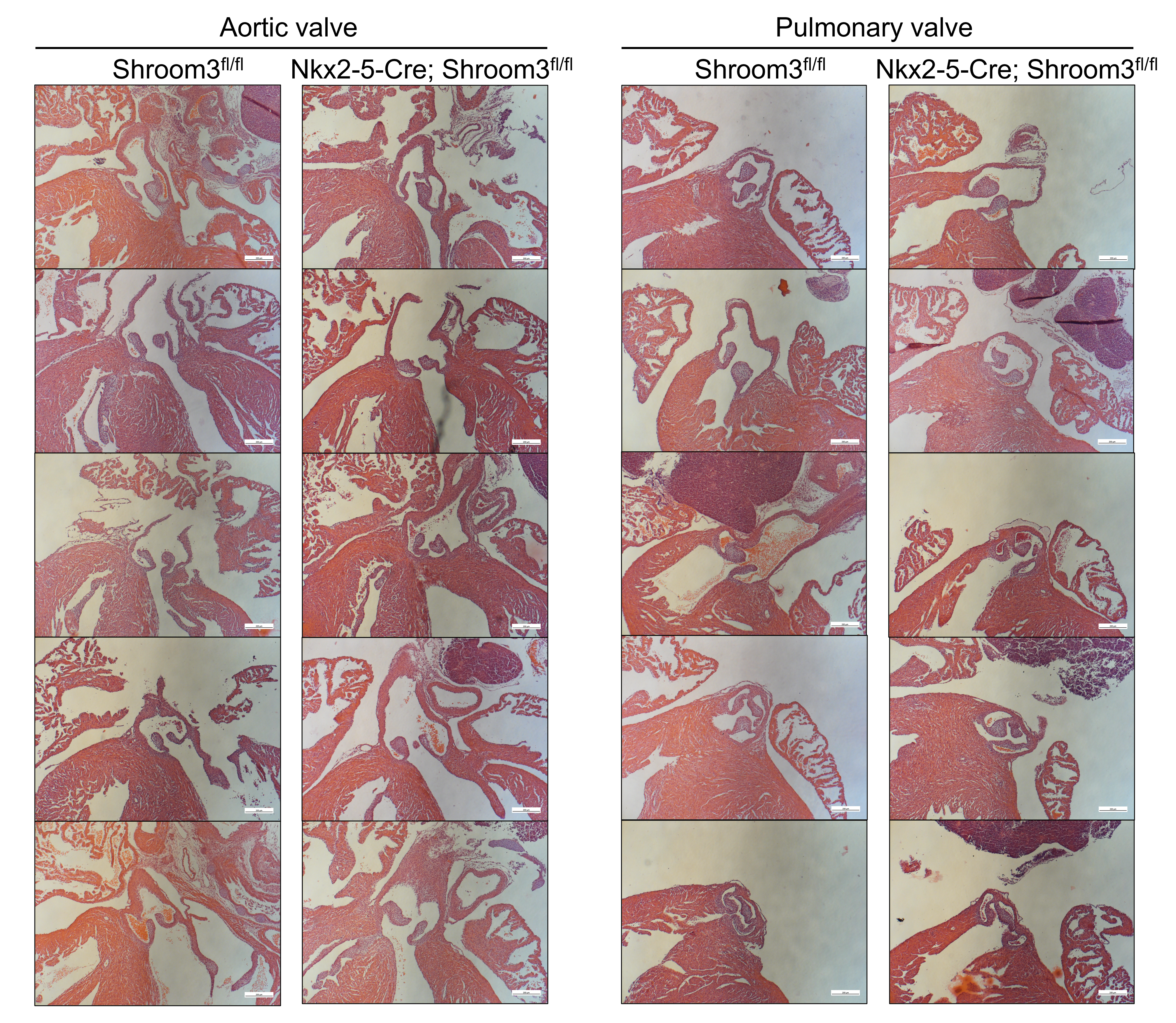

Supplement: S3 Fig — Images of semilunar valves taken from neonate mice from the Nkx2–5-Cre;Shroom3+/fl X Shroom3fl/fl cross. Aortic valves are displayed on the left and pulmonary valves are displayed on the right. Images present differing angles and depths of sectioning. Sectioned in the frontal plane. (TIF) [file pone.0331583.s003.tif]
