# Supplementary material for: Understanding the role of Shroom3 in the developing mouse myocardium
Source: PLoS One. 2025 Sep 8;20(9):e0331583. doi: 10.1371/journal.pone.0331583 (PMC12416694; doi:10.1371/journal.pone.0331583)
Supplement: Attachment — Submitted filename: Response_to_reviewers_auresp_2.docx [file pone.0331583.s005.docx]

August 5^th^, 2025

Dr. Emily Chenette

Editor-in-Chief,

PLOS One

Dear Dr. Chenette:

We are happy to respond to Reviewer #2 and address their concerns about the description of Shroom3 expression in the outflow tract.

Reviewer #2

1. The manuscript describes the Shroom3 expression pattern, which is robust and an important contribution to the manuscript. The manuscript states: “This expression was specific to the myocardium of the atria and ventricles of the heart and was not seen in the outflow tracts or great arteries….” and then later …” Shroom3 expression was seen in the base of the outflow tract“

The staining pattern is clear and there is clearly a sharp drop-off and absence in the great arteries. However, it seems contradictory to say staining is not present in the outflow tract and then later that it is present in the base of the outflow tract. It seems more accurate to state that staining was localized to the base of the outflow tract and absent from the great arteries.

This is an important point given that cardiac neural crest cells are responsible for outflow tract, aortopulmonary, and ventricular septation, and the article hypothesizes (logically) that neural crest cells may be responsible for the septal defects seen in whole body loss of function embryos.

Clarification of this minor point would make the manuscript acceptable for publication

In saying “the base of the outflow tracts”, we were referring to the portion of the heart which surrounds the base of the outflow tracts, rather than the most basal portion of the outflow tract itself. We understand that our description was unclear and we thank you for bringing it to our attention. To make this clearer, we have amended the sentences to read “the myocardium surrounding the base of the outflow tracts”. These changes can be found on page 10, line 219-220; page 11, line 237-238, 254; and page 20, line 475.

We once again thank the reviewers for their attention to detail and their suggestions and insights which have improved the quality of the manuscript.

Sincerely,

Jennifer Carleton

PhD candidate, Drysdale lab

Department of Physiology and Pharmacology, Developmental Biology specialization

Schulich School of Medicine and Dentistry

The University of Western Ontario
